# Evaluation of the association between polymorphisms of *PRM1* and *PRM2* and the risk of male infertility: a systematic review, meta-analysis, and meta-regression

**DOI:** 10.1038/s41598-020-74233-3

**Published:** 2020-10-14

**Authors:** Houshang Nemati, Masoud Sadeghi, Mehri Nazeri, Mohana Mohammadi

**Affiliations:** 1 Fertility and Infertility Research Center, Health Technology Institute, Kermanshah University of Medical Sciences, Kermanshah, Iran; 2 Medical Biology Research Center, Kermanshah University of Medical Sciences, Kermanshah, Iran; 3 Student Research Committee, Kermanshah University of Medical Sciences, Kermanshah, Iran

**Keywords:** Genetics, Molecular medicine

## Abstract

Studies have reported the genetic gives rise to male infertility. The aim of the present meta-analysis was to evaluate the association between *PRM1* (rs737008 and rs2301365) and *PRM2* (rs1646022 and rs2070923) polymorphisms and susceptibility to male infertility. The association between *PRM1* and *PRM2* polymorphisms and the risk of male infertility was evaluated using specific search terms in the Web of Science, Cochrane Library, PubMed, and Scopus databases without language restriction until January 28, 2020. The association was determined by odds ratio (OR) and 95% confidence interval (CI) on five genetic models using Review Manager 5.3 software. The funnel plot analysis and sensitivity analysis were done by the Comprehensive Meta-analysis 2.0 software. Out of 261 records retrieved from the databases, 17 studies were analyzed in the meta-analysis, including the four *PRM* polymorphisms. The pooled results as OR (*P*-value) showed 0.96 (0.44), 1.04 (0.70), 0.94 (0.51), 0.94 (0.48), and 1.03 (0.72) for *PRM1* rs737008 polymorphism and 1.67 (0.0007), 1.73 (0.06), 1.50 (0.007), 1.56 (0.004), and 1.62 (0.33) for *PRM1* rs2301365 polymorphism in allele, homozygous, heterozygous, recessive, and dominant models, respectively. Moreover, the pooled results as OR (*P*-value) showed 1.19 (0.004), 1.15 (0.26), 1.08 (0.70), 1.05 (0.76), and 0.98 (0.82) for *PRM2* rs1646022 and 0.88 (0.04), 0.84 (0.10), 1.05 (0.81), 0.90 (0.24), and 0.80 (0.02) for *PRM2* rs2070923 in allele, homozygous, heterozygous, recessive, and dominant models, respectively. The results showed *PRM1* rs2301365 and *PRM2* rs1646022 polymorphisms were associated with an elevated risk of male infertility and *PRM2* rs2070923 polymorphism had a protective role in infertile men.

## Introduction

Infertility is defined as couples' inability to have a baby after one year of regular unprotected intercourse^1^. Male factor infertility affects up to 50% of couples' infertility and accounts for only 20% of total infertility^2^. Recently, however, the male factor infertility incidence has increased^3,4^. Male infertility is currently assessed through routine analysis according to sperm concentration/number, motility, and sperm morphology. However, there is a significant integration of semen characteristics between fertile and infertile males. In fact, around 15% of patients with male factor infertility according to WHO guidelines^5^ have normal semen parameters^6^. Thus, there are several limitations to routine conventional semen analysis in assessing male infertility, indicating that conventional semen parameters are poor predictors of reproductive outcome and that definitive diagnosis of male infertility cannot be made by routine analysis alone^7^. These limitations have led to the development of advanced methods for the study of sperm function, oxidative stress, fragmentation and DNA packing^8^. Non-obstructive azoospermia and severe oligozoospermia are two of the dominant phenotypes associated with severe spermatogenesis^9^. However, many factors relate to male infertility, like to reproductive tract disorders, chemical exposure, and infection^9^. Genetic factors account for 50% or more of all male infertility etiology, and approximately 7% of men worldwide suffer from infertility^10^. In order to indicate the underlying causes, extensive research has been done on the genetic reasons of male infertility in recent years.

There are two types of protamines (PRMNs), PRMN1 and PRMN2, which are encoded by two genes, *PMN1* and *PMN2*, located on chromosome 16. In human sperm cells, 85% of histones are replaced by PRMN and from DNA in Protect against harmful agents. Altered ratio of histones to proteins has been shown to increase chromatin deficiency in sperm, increasing the risk of DNA damage and male infertility. In addition, an adequate ratio of PRMN1 and PRMN2 (normal 0.8–1.2) is needed for normal sperm function^11^. The expression of these two proteins in the sperm nucleus is approximately equal^12^. The complete translation of PRM1 and PRM2 mRNA happens throughout the elongated spermatids development, occurring in the production of positively charged PRMNs as a result of the high arginine content and this allows for strong binding to negatively charged DNA^13^. It was noticed a significantly diminished level of PRM1 mRNAs in spermatozoa isolated from crossbred Frieswal bulls with poor semen parameters, mostly featured by low progressive motility, in comparison to a group with good semen features^14^ and decreased PRM2 levels have been reported in various studies in infertile patients^15^. PRMs are believed to play a significant role in chromatin aggregation, transcriptional repression, haploid male genome conservation, sperm formation, and offspring production^16^. There were two previous meta-analyses reporting an association between *PRM* polymorphisms and the risk of male infertility including 8 studies^17^ and checking one *PRM* polymorphism and another^9^ included 13 studies with six *PRM* polymorphisms. Therefore, in the present meta-analysis including a meta-regression analysis of 17 studies, we investigated 13 *PRM* polymorphisms and then focused on the association between four functional *PRM1* (rs737008 and rs2301365) and *PRM2* (rs1646022 and *PRM2* rs2070923) polymorphisms and male infertility susceptibility in case–control studies.

## Materials and methods

The meta-analysis was done based on PRISMA statement, and the study question was formulated based on the PICOS framework^18,19^.

Participants (P): Men with infertility

Interventions (I): Prevalence of *PRM1* and *PRM2* polymorphisms

Comparisons (C): Male healthy controls

Outcomes (O): Risk of *PRM1* and *PRM2* polymorphisms

Study design (S): Case–control studies

### Literature search

To search the association of *PRM1* and *PRM2* polymorphisms with the risk of male infertility, one author used the search terms ("male infertility") and (“PRM1” or “PRM2" or “Protamine 1” or “Protamine 2”) and (“gene*” or “variant*” or “polymorphism*” or “single-nucleotide polymorphism”) in the Web of Science, Cochrane Library, PubMed, and Scopus databases without language restriction until January 28, 2020. Another author checked the titles and abstracts to exclude the duplicates and irrelevant records and checked the full-texts of eligible studies. The databases were searched manually by crosschecking the references of original papers, review papers, and previous meta-analyses related to our topic in this meta-analysis to find the possibly missed studies. In addition, among studies retrieved, two previous meta-analyses had reported an association between *PRM* polymorphisms and the risk of male infertility^9,17^. One of them^17^ included 8 studies checking *PRM1* rs2301365 polymorphism and showed an association between this polymorphism and the risk of male infertility just in Caucasians. Another^9^ included 13 studies (11 studies on *PRM1* and 7 studies on *PRM2* polymorphisms) with six *PRM* polymorphisms and showed an association between *PRM1* rs737008, *PRM1* rs2301365, and *PRM2* rs1646022 polymorphisms and the risk of male infertility.

### Inclusion and exclusion criteria

The inclusion criteria included (1) study focus on *PRM1* polymorphisms rs35576928, rs737008, rs35262993, rs2301365, rs140477029, and rs193922261 and also *PRM2* polymorphisms of rs1646022, rs779337774, rs545828790, rs201933708, rs115686767, rs200072135, and rs2070923 with male infertility susceptibility; (2) case–control studies on human beings that the cases were infertile patients with idiopathic infertility and including all subtypes (mainly azoospermia, cryptozoospermia, and oligozoospermia) and the controls were fertile; (3) including the details of genotype or allele frequency of cases and controls; (4) studies with complete full-text, and (5) studies with every language, (6) studies with or without deviation from the Hardy–Weinberg equilibrium (HWE) in controls. The exclusion criteria included (1) studies not concerning the association between *PRM* polymorphisms mentioned above and male infertility susceptibility; (2) animal articles, review studies, meta-analyses, and conference papers or editorial articles; (3) duplicate studies; and (4) studies with irrelevant data.

### Data extraction and verification

The information retrieved from each study is mentioned in Tables 1, 2, and 3, including: (I) the first author’s name, (II) publication year, (III) region of origin and ethnicity, (IV) genotyping methods, (V) number of both cases and controls, (VI) HWE in the controls, (VII) control sources, and (VIII) prevalence of genotypes and alleles. Two authors independently extracted all the data of the studies included in the meta-analysis. In the case of disagreement between the two authors, another author resolved the disagreement by review and discussion.

**Table 1:** Main characteristics of all studies entered to the meta-analysis.

First author, publication year	Country	Ethnicity	No. of patients to controls	Method	Control source
Tanaka, 2003^24^	Japan	Asian	226/270	PCR sequence	PB
Aoki, 2006^25^	USA	Mixed	192/96	PCR sequence	HB
Ravel, 2007^26^	France	Caucasian	281/111	PCR–RFLP and sequence	PB
Gazquez, 2008^27^	Spain	Caucasian	220/101	PCR–RFLP and sequence	PB
Imken, 2009^28^	Morocco	Caucasian	135/160	PCR sequence	PB
Tuttelmann, 2010^29^	Germany	Caucasian	171/77	PCR sequence	PB
Jodar, 2011^23^	Spain and Sweden	Caucasian	156/102 and 53/50	PCR sequence	HB
Venkatesh, 2011^30^	India	Caucasian	100/100	PCR sequence	PB
Grassetti, 2012^31^	Italy	Caucasian	110/53	PCR sequence	HB
He, 2012^32^	China	Asian	304/369	Mass ARRAY	HB
Siasi, 2012^33^	Iran	Caucasian	96/100	PCR–RFLP, PCR–SSCP and PCR sequencing	HB
Yu, 2012^34^	China	Asian	157/37	Mass ARRAY	HB
Jamali, 2016^35^	Iran	Caucasian	130/130	PCR–RFLP	PB
Jiang, 2017^36^	China	Asian	636/442	Mass ARRAY	HB
Aydos, 2018^37^	Turkey	Caucasian	100/100	PCR	HB
Nabi, 2018^38^	Iran	Caucasian	100/100	PCR sequence	HB
Dehghanpour, 2019^39^	Iran	Caucasian	65/65	PCR sequence	HB

*PCR* Polymerase chain reaction, *RFLP* restriction fragment length polymorphism, *SSCP* single-strand conformation polymorphism, *HB* hospital-based, *PB* population-based.

**Table 2:** Prevalence of genotypes and alleles of PRM1 and PRM2 polymorphisms.

First author, publication year	PRM1 polymorphism	Case			Control			Case		Control		HWE*
		CC	CA	AA	CC	CA	AA	C	A	C	A	
Tanaka, 2003^24^	rs737008	125	86	15	129	117	24	336	116	375	165	0.728
Aoki, 2006^25^	rs737008	32	79	81	12	43	41	143	241	67	125	0.889
Ravel, 2007^26^	rs737008	38	131	112	14	51	46	207	355	79	143	0.981
Imken, 2009^28^	rs737008	16	55	64	16	74	70	87	183	106	214	0.578
Tuttelmann, 2010^29^	rs737008	23	63	85	8	28	41	109	233	44	110	0.338
Jodar, 2011a^23^	rs737008	12	64	80	14	41	47	88	224	69	135	0.302
Jodar, 2011b^23^	rs737008	2	28	30	4	20	26	32	74	28	72	0.955
Venkatesh, 2011^30^	rs737008	56	20	24	48	24	28	132	68	120	80	< 0.001
Grassetti, 2012^31^	rs737008	15	55	40	4	29	20	85	135	37	69	0.137
He, 2012^32^	rs737008	161	112	31	209	142	25	434	174	560	192	0.894
Siasi, 2012^33^	rs737008	22	32	42	24	29	47	76	116	77	123	< 0.001
Nabi, 2018^34^	rs737008	33	47	12	21	51	15	123	61	93	81	0.096
Dehghanpour, 2019^35^	rs737008	0	62	3	17	37	11	62	68	71	59	0.232
Ravel, 2007^26^	rs2301365	184	87	10	71	36	4	455	287	178	44	0.829
Gazquez, 2008^27^	rs2301365	114	90	16	68	30	3	318	122	166	36	0.887
Imken, 2009^28^	rs2301365	85	45	5	113	42	5	215	55	268	52	0.652
Jodar, 2011a^23^	rs2301365	88	55	13	60	38	4	231	81	158	46	0.501
Jodar, 2011b^23^	rs2301365	25	27	1	26	17	7	77	29	69	31	0.176
He, 2012^32^	rs2301365		100	17	241	112	16	474	134	594	144	0.517
Yu, 2012^34^	rs2301365	61	70	26	17	19	1	192	122	53	21	0.109
Jamali, 2016^35^	rs2301365	80	39	11	109	20	1	199	61	238	22	0.937
Jiang, 2017^36^	rs2301365	378	229	29	277	144	21	985	287	698	187	0.681
Aydos, 2018^37^	rs2301365	58	38	4	92	8	0	154	46	192	8	0.676

First author, publication year	PRM2 polymorphism	GG	GC	CC	GG	GC	CC	G	C	G	C	
Tanaka, 2003^24^	rs1646022	127	80	19	127	118	24	224	118	372	166	0.645
Aoki, 2006^25^	rs1646022	77	30	85	39	13	44	184	200	91	101	< 0.001
Tuttelmann, 2010^28^	rs1646022	57	66	36	22	28	23	180	138	74	72	0.046
Venkatesh, 2011^30^	rs1646022	100	0	0	98	0	2	200	0	196	4	< 0.001
Grassetti, 2012^31^	rs1646022	30	62	18	18	26	9	122	98	62	44	0.940
Jamali, 2016^35^	rs1646022	4	39	7	93	31	6	207	53	217	43	0.120
Jiang, 2017^36^	rs1646022	35	266	335	47	162	233	336	936	256	478	0.021
Nabi, 2018^38^	rs1646022	31	59	10	36	56	8	121	79	128	72	0.031
Dehghanpour, 2019^39^	rs1646022	29	25	11	20	41	4	83	47	81	49	0.005

First author, publication year	PRM2 polymorphism	CC	CA	AA	CC	CA	AA	C	A	C	A	
Tanaka, 2003^24^	rs2070923	125	82	19	127	118	25	332	120	372	168	0.747
Aoki, 2006^25^	rs2070923	93	27	72	40	12	44	213	171	81	100	< 0.001
Tuttelmann, 2010^29^	rs2070923	78	55	26	38	26	9	211	107	102	44	0.187
Venkatesh, 2011^30^	rs2070923	55	20	25	60	0	40	130	70	120	80	< 0.001
Grassetti, 2012^31^	rs2070923	42	54	14	23	25	5	138	82	71	35	0.628
He, 2012^32^	rs2070923	87	57	162	99	73	204	231	381	271	481	< 0.001
Nabi, 2018^38^	rs2070923	15	57	28	23	34	43	87	113	80	120	0.003
Dehghanpour, 2019^39^	rs2070923	21	22	22	11	26	28	64	66	48	56	0.254

*HWE* Hardy–Weinberg equilibrium.

*P-values of HWE for control group. The study of Jodar et al.^17^ included two studies.

**Table 3:** Prevalence of genotypes and alleles of other PRM1 and PRM2 polymorphisms.

First author, publication year	PRM1 polymorphism	Case			Control			Case		Control	
		GG	GA	AA	GG	GA	AA	G	A	G	A
Aoki, 2006^25^	rs35262993	189	3	0	94	2	0	381	3	190	2
Ravel, 2007^26^	rs35262993	111	0	0	281	0	0	222	0	562	0
Imken, 2009^28^	rs35262993	133	2	0	155	5	0	315	5	271	2
Tuttelmann, 2010^29^	rs35262993	167	4	0	75	2	0	338	4	152	2
Grassetti, 2012^31^	rs35262993	109	1	0	53	0	0	106	1	119	0
He, 2012^32^	rs35262993	292	1	0	373	1	0	585	1	747	1

First author, publication year	PRM1 polymorphism	CC	CT	TT	CC	CT	TT	C	T	C	T
Jodar, 2011a^23^	rs140477029	155	1	0	102	0	0	311	1	204	0
Dehghanpour, 2019^39^	rs140477029	65	0	0	65	0	0	130	0	130	0

First author, publication year	PRM1 polymorphism	GG	GT	TT	GG	GT	TT	G	T	G	T
Aoki, 2006^25^	rs35576928	189	3	0	94	2	0	381	3	190	2
Ravel, 2007^26^	rs35576928	111	0	0	278	3	0	222	0	559	3
Tuttelmann, 2010^29^	rs35576928	167	4	0	75	2	0	338	4	152	2
Jodar, 2011a^23^	rs35576928	155	1	0	102	0	0	311	1	204	0
Jodar, 2011b^23^	rs35576928	52	1	0	49	1	0	104	1	99	1
Grassetti, 2012^31^	rs35576928	110	0	0	52	1	0	220	0	105	1
He, 2012^32^	rs35576928	328	45	0	256	47	0	701	45	559	47
Aydos, 2018^37^	rs35576928	100	0	0	100	0	0	200	0	200	0
Nabi, 2018^38^	rs35576928	92	0	0	87	0	0	182	0	174	0
Zeyadi, 2019^22^	rs35576928	9	6	0	9	1	0	24	0	19	1
Dehghanpour, 2019^39^	rs35576928	65	0	0	65	0	0	130	0	130	0

First author, publication year	PRM1 polymorphism	GG	GC	CC	GG	GC	CC	G	C	G	C
Ravel, 2007^26^	rs193922261	111	0	0	281	0	0	222	0	562	0
Imken, 2009^28^	rs193922261	134	1	0	160	0	0	269	1	320	0

First author, publication year	PRM2 polymorphism	CC	CT	TT	CC	CT	TT	C	T	C	T
Siasi, 2012^33^	rs779337774	100	0	0	100	0	0	200	0	200	0
Aydos, 2018^37^	rs779337774	98	2	0	100	0	0	198	2	200	0
Nabi, 2018^38^	rs779337774	92	0	0	87	0	0	184	0	174	0
Zeyadi, 2019^22^	rs779337774	33	3	4	9	1	0	69	11	19	1

First author, publication year	PRM2 polymorphism	GG	GA	AA	GG	GA	AA	G	A	G	A
Nabi, 2018^38^	rs545828790	92	0	0	84	3	0	184	0	171	3
Dehghanpour, 2019^39^	rs545828790	65	0	0	65	0	0	130	0	130	0

First author, publication year	PRM2 polymorphism	GG	GC	CC	GG	GC	CC	G	C	G	C
Grassetti, 2012^31^	rs201933708	110	0	0	52	1	0	220	0	105	1
Nabi, 2018^38^	rs201933708	92	0	0	85	2	0	184	0	172	2
Dehghanpour, 2019^39^	rs201933708	65	0	0	61	4	0	130	0	126	4

First author, publication year	PRM2 polymorphism	CC	CT	TT	CC	CT	TT	C	T	C	T
Nabi, 2018^38^	rs115686767	92	0	0	85	2	0	184	0	172	2
Dehghanpour, 2019^39^	rs115686767	65	0	0	61	4	0	130	0	126	4
Aoki, 2006^25^	rs200072135	191	1	0	95	1	0	383	1	191	1
Imken, 2009^28^	rs200072135	135	0	0	159	1	0	170	0	319	1
Jodar, 2011a^23^	rs200072135	111	0	0	49	1	0	222	0	99	1

The study of Jodar et al.^17^ included two studies.

### Statistical analysis

The evaluation of the strength of association between *PRM1* and *PRM2* polymorphisms and male infertility risk was performed by odds ratio (OR) and 95% confidence interval (CI). Review Manager 5.3 software was applied to calculate the summary ORs based on five genetic models (allele, heterozygous, homozygous, recessive, and dominant). In this state, the statistical significance of pooled results was illustrated with the Z-test. *P*-value < 0.05 was considered statistically significant. In addition, heterogeneity across the studies was estimated by the Chi-square-based Q test^20^. If the *P*_*h*_ or *P*_heterogeneity_ was > 0.10 and heterogeneity or I^2^ < 50%, showing lack of heterogeneity between studies, we should use the fixed-effects model, but conversely, we used the random-effects model^21^.

The thirteen polymorphisms were assessed for the association with susceptibility to male infertility based on five genetic models. Among them, four polymorphisms were included in the meta-analysis: *PRM1* (rs737008 and rs2301365) and *PRM2* (rs1646022 and rs2070923). The prevalence rates of CC (wild-type homozygote), CA (heterozygote), and AA genotype (rare homozygote) were calculated for *PRM1* rs737008, *PRM1* rs2301365, and *PRM2* rs2070923 polymorphisms. Further, the GG (wild-type homozygote), GC (heterozygote), and CC (rare homozygote) were calculated for *PRM2* rs1646022 polymorphism. Subgroup analyses were further performed based on ethnicity, method, and control source. A sensitivity analysis was conducted in which the studies with deviation from HWE in the controls were deleted. A meta-regression analysis was performed to detect the confounding factors affecting the pooled results by IBM SPSS 22.0 software. In addition, sensitivity analyses, including “one remove study” and “cumulative analysis”, were conducted each time on previous analyses to determine the stability of the pooled results. Funnel plots and Egger’s liner regression test were used to examine the publication bias. The funnel plot analysis and sensitivity analysis were done by Comprehensive Meta-analysis 2.0 software.

## Results

**Figure 1:**
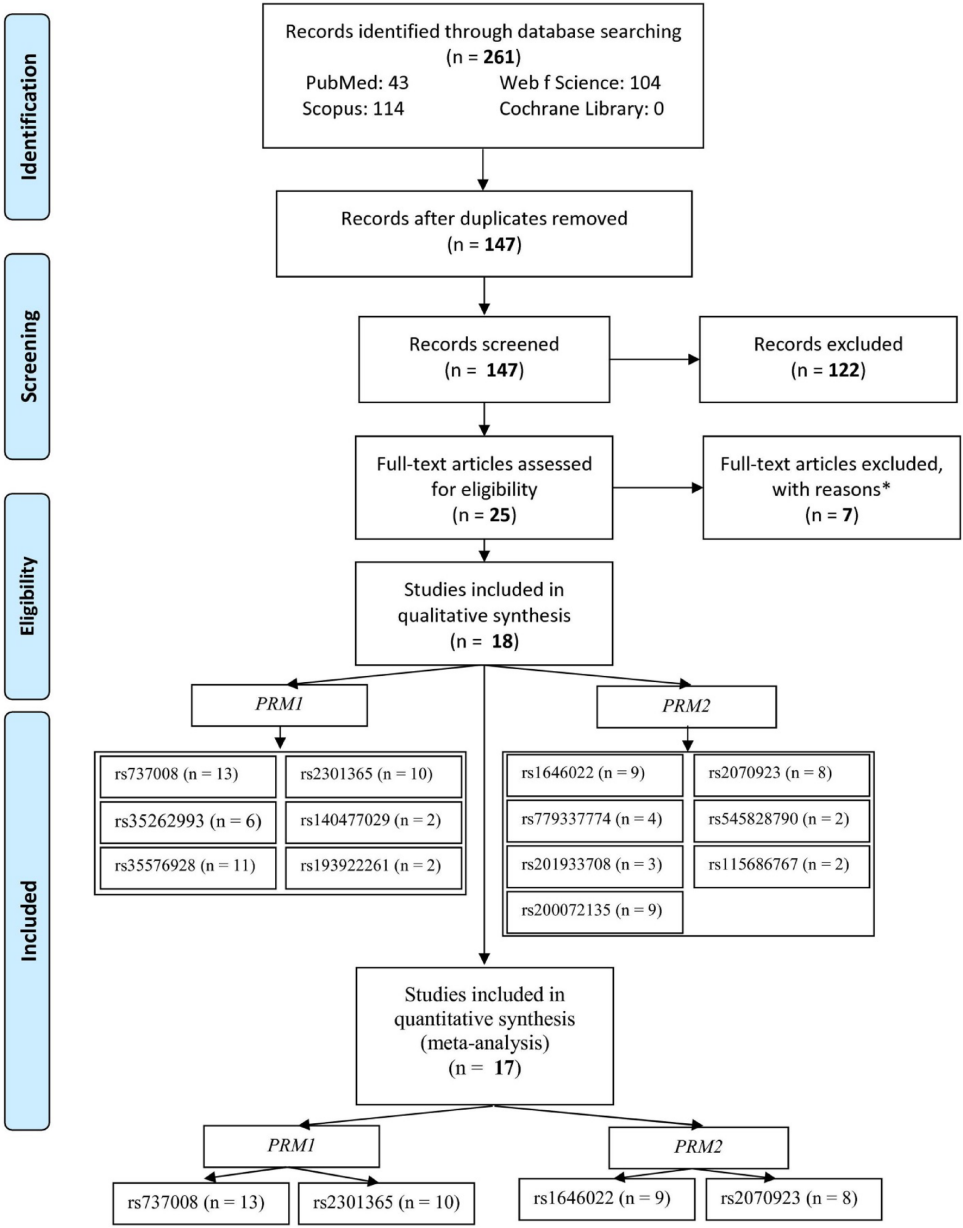
Flow-chart of the study selection. One of articles^23^ included two studies.

Out of 261 records retrieved in the databases, 25 articles including full-texts were evaluated for eligibility after excluding the duplicates and irrelevant records (Fig. 1). Among these full-texts, 7 of them were excluded with reasons (2 meta-analyses, 2 reviews, 1 animal study, and 2 studies with no control groups). Therefore, 18 studies were included in the systematic review, from which one study^22^ was excluded because it did not include four eligible polymorphisms. Finally, 17 studies including four polymorphisms of *PRM1* rs737008, *PRM1* rs2301365, *PRM2* rs1646022, and *PRM2* rs2070923 were analyzed in the meta-analysis. One study^23^ checked the rs737008 and rs2301365 polymorphisms in two different populations (13 for polymorphism of *PRM1* rs737008, 10 for *PRM1* rs2301365, 9 for *PRM2* rs1646022, and 8 for *PRM2* rs2070923).

Table 1 presentations the features of studies entered to the meta-analysis. The studies^23–39^ were published from 2003 to 2019. Twelve studies^23,26–31,33,35,37–39^ were reported in Caucasian, four studies^24,32,34,36^ in Asian, and one^25^ in mixed ethnicities. The genotyping method was PCR-based in fourteen studies^23–31,33,35,37–39^ and Mass ARRAY in three studies^32,34,36^. The source of controls was hospital-based in ten studies^25,31–33,33,34,36–39^ and population-based in seven studies^24,26–30,35^.

Tables 2 and 3 show the prevalence of the genotypes and alleles of *PRM1* and *PRM2* polymorphisms. We included four polymorphisms (*PRM1* rs737008, *PRM1* rs2301365, *PRM2* rs1646022, and *PRM2* rs2070923) in the meta-analysis mentioned in Table 2. The other polymorphisms mentioned (*PRM1* rs35262993, rs140477029, rs35576928, and rs193922261 polymorphisms and *PRM2* rs779337774, rs545828790, rs201933708, rs115686767, and rs200072135 polymorphisms) in Table 3 were excluded from the meta-analysis because a lot of studies had no mutation or the percentage of mutation was very low. The *P*-values of HWE were less than 0.05 for the controls of *PRM1* rs737008 polymorphism in two studies^30,33^, *PRM2* rs1646022 in six studies^25,29,30,36,38,39^, and *PRM2* rs2070923 in four studies^25,30,32,38^.

**Figure 2:**
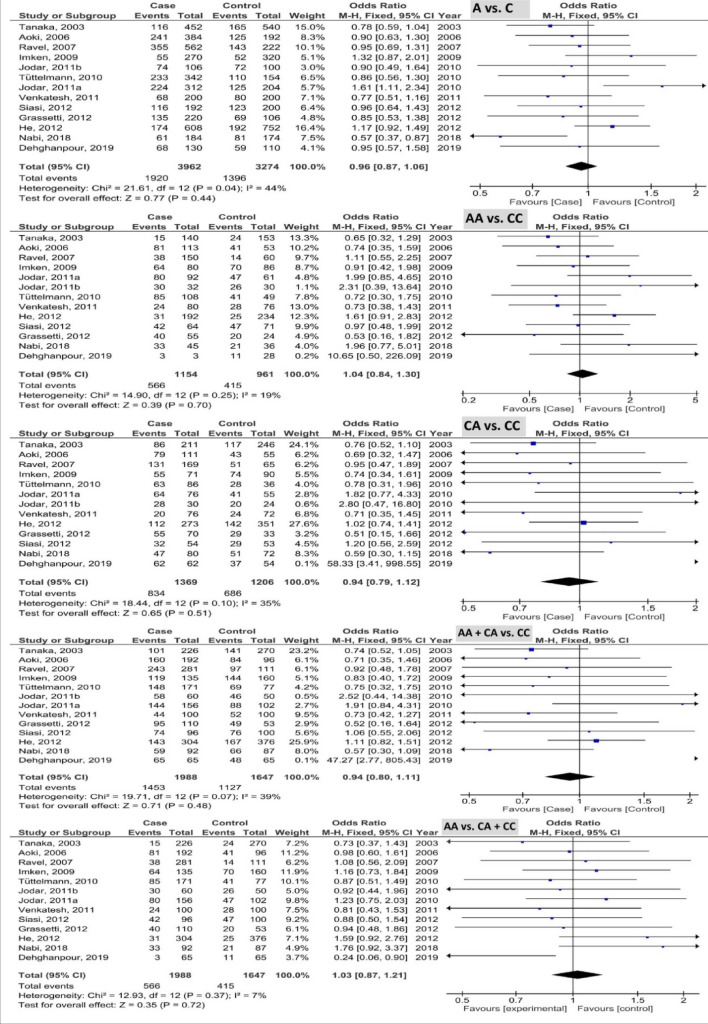
Forest plot of analysis of PRM1 rs737008 polymorphism based on five genetic models.

The pooled results of *PRM1* rs737008 polymorphism based on five genetic models are illustrated in Fig. 2. The pooled results as OR (985%CI; *P*-value) showed 0.96 (0.87, 1.06; 0.44) with I^2^ = 44% (P_heterogeneity_ or P_h_ = 0.04), 1.04 (0.84, 1.30; 0.70) with I^2^ = 19% (P_h_ = 0.25), 0.94 (0.79, 1.12; 0.51) with I^2^ = 35% (P_h_ = 0.10), 0.94 (0.80, 1.11; 0.48) with I^2^ = 39% (P_h_ = 0.07), and 1.03 (0.87, 1.21; 0.72) with I^2^ = 7% (P_h_ = 0.37) in the allele, homozygous, heterozygous, recessive, and dominant models, respectively. Based on the results, this polymorphism was not associated with the male infertility susceptibility.

**Figure 3:**
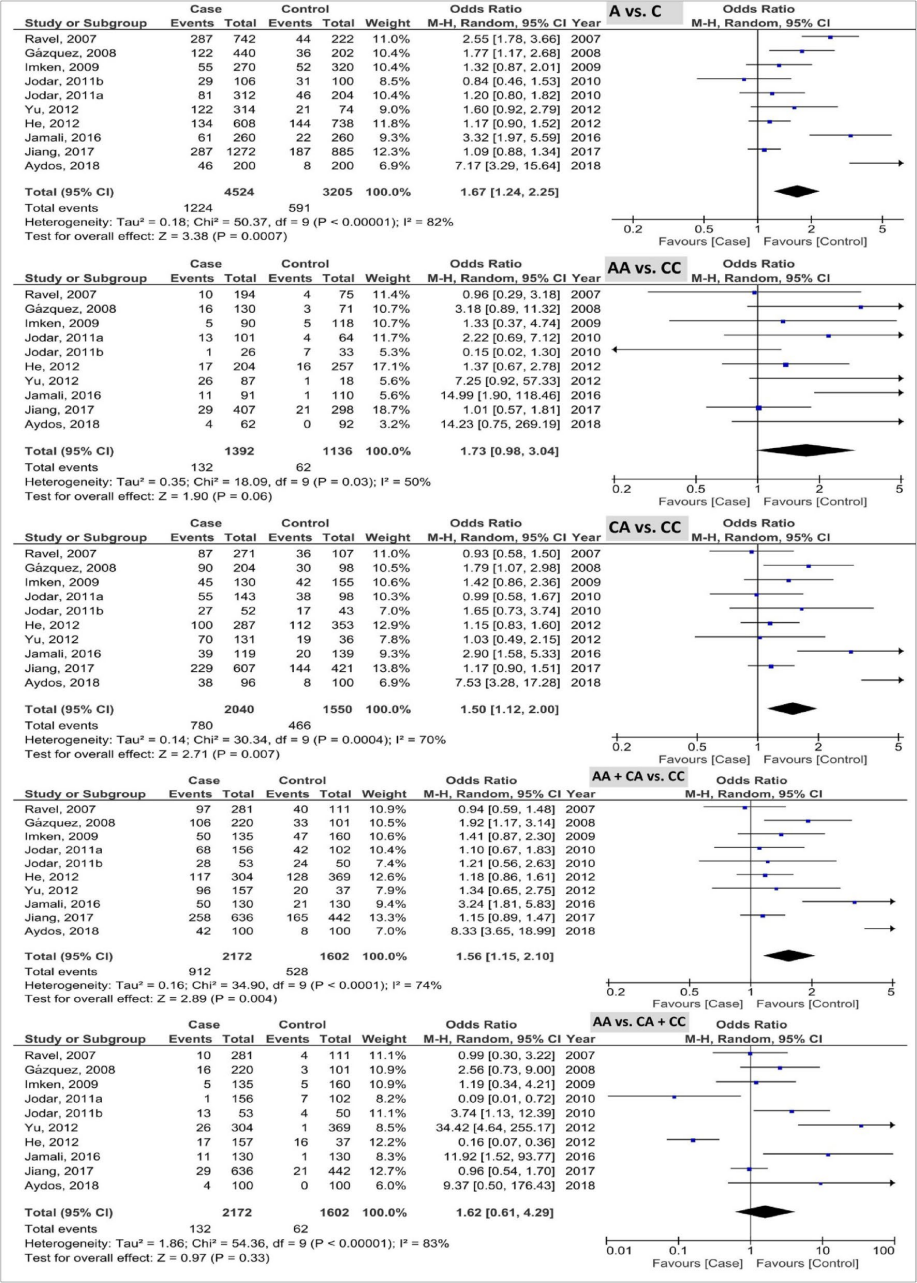
Forest plot of analysis of PRM1 rs2301365 polymorphism based on five genetic models.

The pooled results of *PRM1* rs2301365 polymorphism based on five genetic models are indicated in Fig. 3. The pooled results as OR (95% CI; *P*-value) showed the 1.67 (1.24, 2.25; 0.0007) with I^2^ = 82% (P_h_ < 0.00001), 1.73 (0.98, 3.04; 0.06) with I^2^ = 50% (P_h_ = 0.03), 1.50 (1.12, 2.00; 0.007) with I^2^ = 70% (P_h_ = 0.0004), 1.56 (1.15, 2.10; 0.004) with I^2^ = 74% (P_h_ < 0.0001), and 1.62 (0.61, 4.29; 0.33) with I^2^ = 83% (P_h_ < 0.00001) in the allele, homozygous, heterozygous, recessive, and dominant models, respectively. Based on the results, C allele and CA genotype of *PRM1* rs2301365 polymorphism were associated with the elevated risk of male infertility.

**Figure 4:**
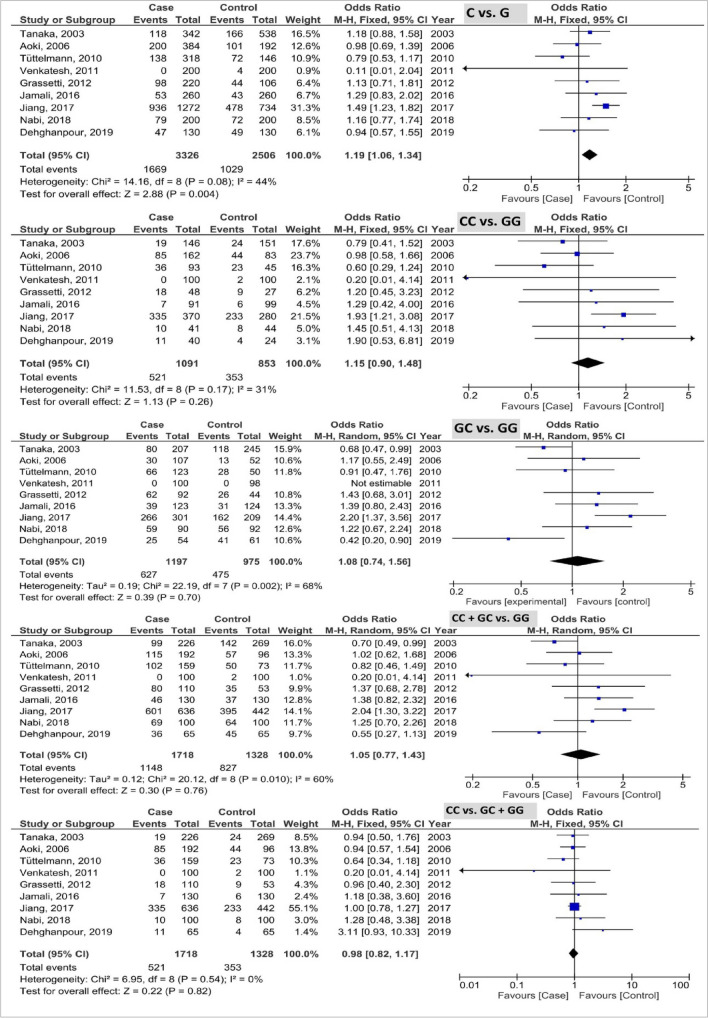
Forest plot of analysis of PRM2 rs1646022 polymorphism based on five genetic models.

The pooled results of *PRM2* rs1646022 polymorphism based on five genetic models are shown in Fig. 4. The pooled results as OR (95% CI; *P*-value) showed the 1.19 (1.06, 1.34; 0.004) with I^2^ = 44% (P_h_ = 0.08), 1.15 (0.90, 1.48; 0.26) with I^2^ = 31% (P_h_ = 0.17), 1.08 (0.74, 1.56; 0.70) with I^2^ = 68% (P_h_ = 0.002), 1.05 (0.77, 1.43; 0.76) with I^2^ = 60% (P_h_ = 0.010), and 0.98 (0.82, 1.17; 0.82) with I^2^ = 0% (P_h_ = 0.54) in the allele, homozygous, heterozygous, recessive, and dominant models, respectively. Based on the results, the G allele of *PRM2* rs1646022 polymorphism was associated with the elevated risk of male infertility.

**Figure 5:**
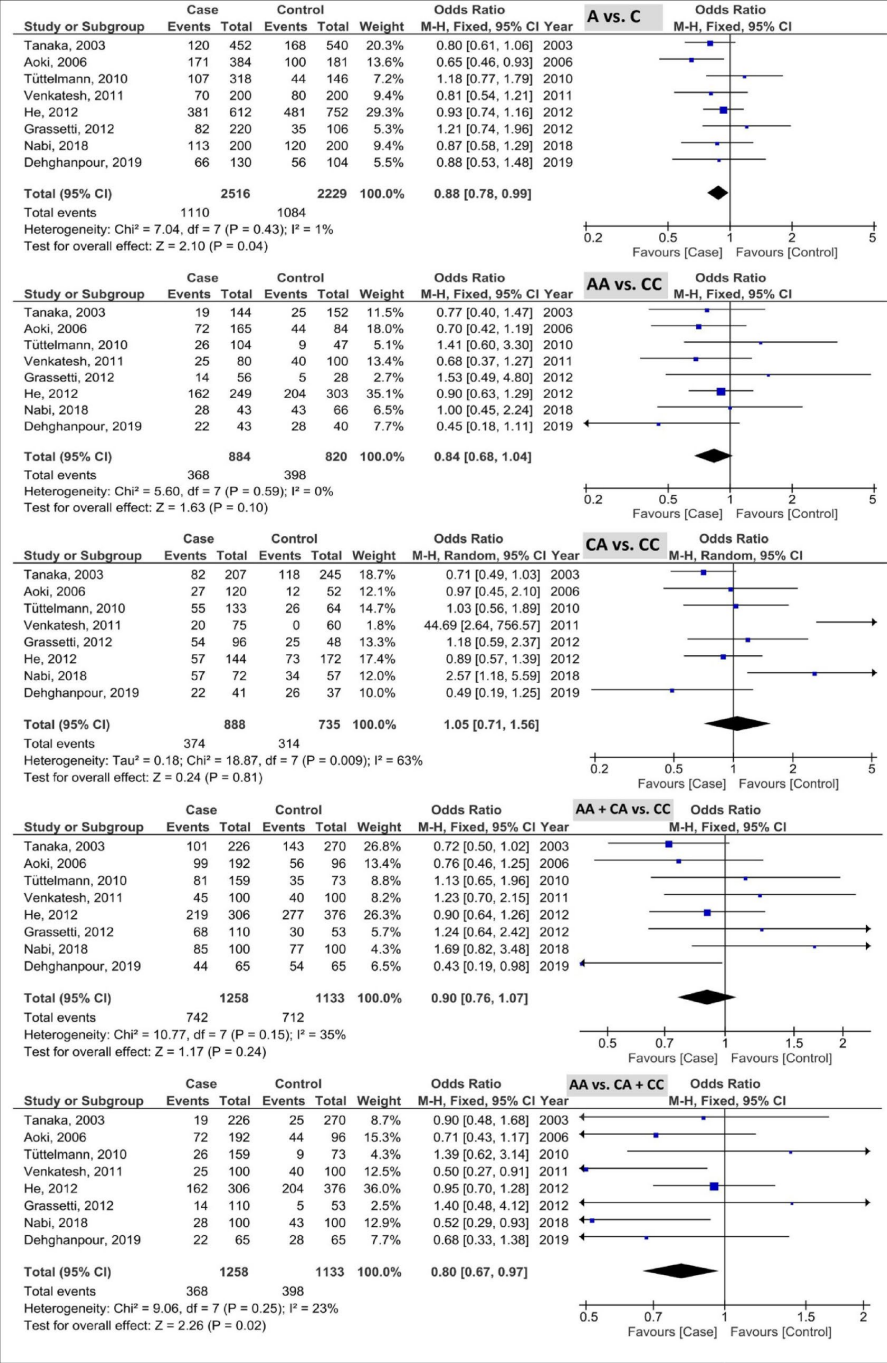
Forest plot of analysis of PRM2 rs2070923 polymorphism based on five genetic models.

The pooled results of *PRM2* rs2070923 polymorphism based on five genetic models are demonstrated in Fig. 5. The pooled results as OR (95% CI; *P*-value) showed the 0.88 (0.78, 0.99; 0.04) with I^2^ = 1% (P_h_ = 0.43), 0.84 (0.68, 1.04; 0.10) with I^2^ = 0% (P_h_ = 0.59), 1.05 (0.71, 1.56; 0.81) with I^2^ = 63% (P_h_ = 0.009), 0.90 (0.76, 1.07; 0.24) with I^2^ = 35% (P_h_ = 015), and 0.80 (0.67, 0.97; 0.02) with I^2^ = 23% (P_h_ = 0.25) in the allele, homozygous, heterozygous, recessive, and dominant models, respectively. Based on the results, the C allele and CC genotype of *PRM2* rs2070923 polymorphism were associated with the reduced risk of male infertility.

### Subgroup analysis

The results of subgroup analysis for *PRM1* rs737008, *PRM1* rs2301365, *PRM2* rs2070923, and *PRM2* rs1646022 polymorphisms are shown in Table 4. The AA + CA genotype in the studies with population-based controls was associated with the reduced risk of male infertility (OR 0.77; 95% CI 0.60, 0.98; *P* = 0.04) without heterogeneity. With regard to *PRM1* rs2301365 polymorphism, the C allele and CA genotype in the Caucasian ethnicity were associated with the elevated risk of male infertility (OR 1.96; 95% CI 1.29, 2.97; *P* = 0.002 and OR 1.79; 95% CI 1.13, 2.83; *P* = 0.01, respectively). Also, the C allele (OR 1.59; 95% CI 1.15, 2.20; *P* = 0.005) and CC (OR 1.44; 95% CI 1.02, 2.03; *P* = 0.04) and CA (OR 1.39; 95% CI 1.01, 1.92; *P* = 0.04) genotypes in the studies with hospital-based controls were associated with the elevated risk of male infertility. For *PRM1* rs2301365 polymorphism, the C allele and CA genotype in the studies with PCR-based method were associated with the elevated risk of male infertility (OR 1.96; 95% CI 1.29, 2.97; *P* = 0.002 and OR 1.79; 95% CI 1.13, 2.83; *P* = 0.01, respectively). About *PRM2* rs2070923 polymorphism, the G allele had an elevated risk in male infertility compared to male fertility (OR 1.38; 95% CI 1.18, 1.63; *P* < 0.0001), which was similar to the G allele (OR 1.26; 95% CI 1.09, 1.46; *P* = 0.001) and GG genotype (OR 1.43; 95% CI 1.06, 1.94; *P* = 0.02) in the studies with hospital-based controls. With regard to mass ARRAY, the G allele (OR 1.49; 95% CI 1.23, 1.82; *P* < 0.0001) and GG (OR 1.93; 95% CI 1.21, 3.08; *P* = 0.006) and GC (OR 2.20; 95% CI 1.37, 3.56; *P* = 0.001) genotypes had an elevated risk in male infertility compared to male fertility. As for *PRM2* rs1646022 polymorphism, the CC genotype was associated with a reduced risk of male infertility (OR 0.69; 95% CI 0.51, 0.94; *P* = 0.02) in the Caucasian ethnicity and C allele (OR 0.65; 95% CI 0.46, 0.93; *P* = 0.02) in the mixed ethnicity. Further, the C allele (OR 0.86; 95% CI 0.74, 0.99; *P* = 0.04) and CC genotype (OR 0.72; 95% CI 0.57, 0.92; *P* = 0.009) in the PCR-based method had a reduced risk of male infertility.

**Table 4:** Subgroup analysis for PRM1 rs737008, PRM1 rs2301365, PRM2 rs2070923, and PRM2 rs1646022 polymorphisms.

PRM1 rs737008	A vs. C	AA vs. CC	CA vs. CC	AA + CA vs. CC	AA vs. CA + CC
	OR (95% CI), *P*, I^2^, P_h_	OR (95% CI), *P*, I^2^, P_h_	OR (95% CI), *P*, I^2^, P_h_	OR (95% CI), *P*, I^2^, P_h_	OR (95% CI), *P*, I^2^, P_h_
Total (13)	0.96 (0.87, 1.06), 0.44, 44, 0.04	1.05 (0.84, 1.31), 0.66, 19, 0.25	0.94 (0.79, 1.12), 0.51, 35, 0.10	0.94 (0.80, 1.11), 0.48, 39, 0.07	1.03 (0.87, 1.21), 0.72, 7, 0.37
Ethnicity					
Asian (2)	0.96, (0.65, 1.43), 0.86, 78, 0.03	1.04 (0.43,2.55), 0.93, 75, 0.04	0.90 (0.71, 1.15), 0.40, 30, 0.23	0.92 (0.61, 1.37), 0.67, 66, 0.09	1.10 (0.51, 2.38), 0.80, 68, 0.08
Caucasian (10)	0.96 (0.84,1.09), 0.50, 47, 0.05	1.08 (0.82, 1.42), 0.60, 10, 0.35	1.04 (0.80, 1.34), 0.79, 47, 0.05	0.98 (0.78, 1.25), 0.89, 46, 0.06	1.01 (0.84, 1.23), 0.90, 5, 0.40
Mixed (1)	0.92 (0.68, 1.23), 0.57	0.74 (0.35, 1.59), 0.44	0.69 (0.32, 1.47), 0.34	0.71 (0.35, 1.46), 0.36	0.98 (0.60, 1.61), 0.93
Control source					
HB (8)	0.97 (0.79, 1.20), 0.81, 54, 0.03	1.32 (0.97, 1.78), 0.07, 22, 0.25	1.06 (0.67, 1.66), 0.82, 57, 0.02	1.09 (0.60, 1.98), 0.78, 63, 0.01	1.09 (0.88, 1.35), 0.42, 32, 0.17
PB (5)	0.89 (0.76, 1.05), 0.16, 17, 0.31	0.81 (0.59, 1.12), 0.20, 0, 0.83	0.78 (0.60, 1.01), 0.06, 0, 0.98	**0.77 (0.60, 0.98), 0.04, 0, 0.98**	0.95 (0.73, 1.22), 0.67, 0, 0.77
Method					
PCR-based (12)	0.92 (0.82, 1.03), 0.15, 40, 0.07	0.97 (0.76, 1.24), 0.81, 10, 0.35	0.91 (0.74, 1.12), 0.39, 38, 0.09	0.88 (0.73, 1.07), 0.21, 36, 0.10	0.99 (0.83, 1.17), 0.88, 0, 0.50
Mass ARRAY (1)	1.17 (0.92, 1.49), 0.20	1.61 (0.91, 2.83), 0.10	1.02 (0.74, 1.41), 0.89	1.11 (0.82, 1.51), 0.49	1.59 (0.92, 2.76), 0.10

PRM1 rs2301365	A vs. C	AA vs. CC	CA vs. CC	AA + CA vs. CC	AA vs. CA + CC
	OR (95% CI), *P*, I^2^, P_h_	OR (95% CI), *P*, I^2^, P_h_	OR (95% CI), *P*, I^2^, P_h_	OR (95% CI), *P*, I^2^, P_h_	OR (95% CI), *P*, I^2^, P_h_
Total (10)	**1.67 (1.24, 2.25), 0.0007, 82, < 0.00001**	1.73 (0.98, 3.04), 0.06, 50, 0.03	**1.50 (1.12, 2.00), 0.007, 70, 0.0004**	**1.56 (1.15, 2.10), 0.004, 74, < 0.0001**	1.62 (0.61, 4.29), 0.33, 83, < 0.00001
Ethnicity					
Asian (3)	1.15 (0.98, 1.35), 0.08, 0, 0.43	1.34 (0.87, 2.05), .0.19, 42, 0.18	1.15 (0.94, 1.40), 0.16, 0, 0.95	1.17 (0.97, 1.41), 0.10, 0, 0.92	1.41 (0.15, 13.18), 0.76, 94, < 0.00001
Caucasian (7)	**1.96 (1.29, 2.97), 0.002, 82, < 0.0001**	1.96 (0.82, 4.70), 0.13, 55, 0.04	**1.79 (1.13, 2.83), 0.01, 77, 0.0003**	**1.82 (1.13, 2.93), 0.01, 80, < 0.0001**	1.82 (0.71, 4.68), 0.21, 61, 0.02
Control source					
HB (8)	**1.59 (1.15,2.20), 0.005, 82, < 0.00001**	**1.44 (1.02, 2.03), 0.04, 45, 0.08**	**1.39 (1.01, 1.92), 0.04, 70, 0.001**	**1.44 (1.04, 1.98), 0.03, 73, 0.0006**	1.38 (0.45, 4.23), 0.57, 85, < 0.00001
PB (2)	2.06 (0.83, 5.10), 0.12, 86, 0.007	3.91 (0.33, 45.85), 0.28, 76, 0.04	0.99 (0.99, 3.99), 0.05, 68, 0.08	2.11 (0.93, 4.75), 0.07, 78, 0.03	3.28 (0.32, 34.09), 0.32, 74, 0.05
Method					
PCR-based (7)	**1.96 (1.29, 2.97), 0.002, 82, < 0.0001**	1.96 (0.82, 4.70), 0.13, 55, 0.04	**1.79 (1.13, 2.83), 0.01, 77, 0.0003**	**1.82 (1.13, 2.93), 0.01, 80, < 0.0001**	1.82 (0.71, 4.68), 0.21, 61, 0.02
Mass ARRAY (3)	1.15 (0.98, 1.35), 0.08, 0, 0.43	1.34 (0.87, 2.05), 0.19, 42, 0.18	1.15 (0.94, 1.40), 0.16, 0, 0.95	1.17 (0.97, 1.41), 0.10, 0, 0.92	1.41 (0.15, 13.18), 0.76, 94, < 0.00001

PRM2 rs1646022	C vs. G	CC vs. GG	GC vs. GG	CC + GC vs. GG	CC vs. GC + GG
	OR (95% CI), *P*, I^2^, P_h_	OR (95% CI), *P*, I^2^, P_h_	OR (95% CI), *P*, I^2^, P_h_	OR (95% CI), *P*, I^2^, P_h_	OR (95% CI), *P*, I^2^, P_h_
Total (9)	**1.19 (1.06, 1.34), 0.004, 44, 0.08**	1.15 (0.90, 1.48), 0.26, 31, 0.17	1.08 (0.74, 1.56), 0.70, 68, 0.002	1.05 (0.77, 1.43), 0.76, 0.60, 0.01	0.98 (0.82, 1.17), 0.82, 0, 0.54
Ethnicity					
Asian (2)	**1.38 (1.18, 1.63), < 0.0001, 42, 0.19**	1.27 (0.53, 3.05), 0.59, 79, 0.03	1.21 (0.38, 3.85), 0.75, 93, 0.0001	1.18 (0.41, 3.39), 0.76, 92, 0.0003	0.99 (0.79, 1.24), 0.93, 0, 0.85
Caucasian (6)	1.02 (0.84, 1.24), 0.86, 12, 0.34	0.99 (0.65, 1.50), 0.94, 0, 0.44	1.04 (0.78, 1.39), 0.78, 48, 0.10	1.03 (0.79, 1.35), 0.81, 27, 0.23	0.98 (0.67, 1.43), 0.90, 27, 0.23
Mixed (1)	0.98 (0.69, 1.39), 0.91	0.98 (0.58, 1.66), 0.94	1.17 (0.55, 2.49), 0.69	1.02 (0.62, 1.68), 0.93	0.94 (0.57, 1.54), 0.80
Control source					
HB (5)	**1.26 (1.09, 1.46), 0.001, 39, 0.16**	**1.43 (1.06, 1.94), 0.02, 0, 0.43**	1.18 (0.69, 2.01), 0.55, 70, 0.009	1.19 (0.79, 1.80), 0.41, 61, 0.04	1.03 (0.84, 1.27), 0.74, 0, 0.45
PB (4)	1.05 (0.86, 1.29), 0.62, 48, 0.12	0.74 (0.48, 1.14), 0.18, 0, 0.57	0.92 (0.59, 1.44), 0.71, 55, 0.11	0.84 (0.65, 1.09), 0.20, 44, 0.15	0.79 (0.53, 1.18), 0.25, 0, 0.56
Method					
PCR-based (9)	1.05 (0.91, 1.21), 0.52, 0, 0.48	0.94 (0.70, 1.26), 0.68, 0, 0.65	0.91 (0.73, 1.13), 0.39, 47, 0.08	0.91 (0.75, 1.11), 0.37, 31, 0.18	0.96 (0.73, 1.26), 0.75, 0, 0.44
Mass ARRAY (1)	**1.49 (1.23, 1.82), < 0.0001**	**1.93 (1.21, 3.08), 0.006**	**2.20 (1.37, 3.56), 0.001**	**2.04 (1.30, 3.22), 0.002**	1.00 (0.78, 1.27), 0.99

PRM2 rs2070923	A vs. C	AA vs. CC	CA vs. CC	AA + CA vs. CC	AA vs. CA + CC
	OR (95% CI), *P*, I^2^, P_h_	OR (95% CI), *P*, I^2^, P_h_	OR (95% CI), *P*, I^2^, P_h_	OR (95% CI), *P*, I^2^, P_h_	OR (95% CI), *P*, I^2^, P_h_
Total (8)	**0.88 (0.78, 0.99), 0.04, 1, 0.43**	0.84 (0.68, 1.04), 0.10, 0, 0.59	1.05 (0.71, 1.56), 0.81, 0.63, 0.009	0.90 (0.76, 1.07), 0.24, 35, 0.15	**00.80 (0.67, 0.97), 0.02, 23, 0.25**
Ethnicity					
Asian (2)	0.88 (0.74, 1.04), 0.13, 0, 0.41	0.87 (0.64, 1.19), 0.38, 0, 0.68	0.78 (0.58, 1.03), 0.08, 0, 0.44	0.81 (0.63, 1.03), 0.09, 0, 0.37	1.68 (0.44, 6.44), 0.45, 80, 0.03
Caucasian (5)	0.96 (0.79, 1.17), 0.71, 0, 0.60	0.86 (0.60, 1.23), 0.40, 19, 0.29	1.40 (0.66, 3.00), 0.38, 73, 0.005	1.11 (0.83, 1.47), 0.48, 40, 0.16	**0.69 (0.51, 0.94), 0.02, 39, 0.16**
Mixed (1)	**0.65 (0.46, 0.93), 0.02**	0.70 (0.42, 1.19), 0.19	0.97 (0.45, 2.10), 0.93	0.76 (0.46, 1.25), 0.28	0.71 (0.43, 1.17), 0.17
Control source					
HB (5)	0.88 (0.75, 1.02), 0.10, 13, 0.33	0.84 (0.65, 1.08), 0.17, 0, 0.45	1.06 (0.68, 1.67), 0.79, 52, 0.08	0.91 (0.72, 1.14), 0.41, 46, 0.12	0.81 (0.65, 1.01), 0.06, 17, 0.31
PB (3)	0.88 (0.72, 1.07), 0.19, 17, 0.30	0.84 (0.57, 1.24), 0.38, 0, 0.38	1.26 (0.46, 3.41), 0.65, 80, 0.006	0.89 (0.69, 1.16), 0.41, 41, 0.19	0.82 (0.46, 1.44), 0.48, 53, 0.12
Method					
PCR-based (7)	**0.86 (0.74, 0.99), 0.04, 10, 0.35**	0.80 (0.61, 1.05), 0.10, 0, 0.51	1.11 (0.68, 1.83), 0.67, 68, 0.004	0.90 (0.74, 1.10), 0.32, 44, 0.10	**0.72 (0.57, 0.92), 0.009, 16, 0.31**
Mass ARRAY (1)	0.93 (0.74, 1.16), 0.52	0.90 (0.63, 1.29), 0.58	0.89 (0.57, 1.39), 0.61	0.90 (0.64, 1.26), 0.54	0.95 (0.70, 1.28), 0.73

*PCR* Polymerase chain reaction, *HB* hospital-based, *PB* population-based. Bold numbers indicate statistically significant differences.

### Meta-regression analysis

The results of meta-regression analysis for four polymorphisms based on publication year are shown in Table 5. The publication year could be a cofounding factor for *PRM1* rs737008, *PRM1* rs2301365, and *PRM2* rs1646022 polymorphisms.

**Table 5:** Meta-regression analysis for PRM1 rs737008, PRM1 rs2301365, PRM2 rs2070923, and PRM2 rs1646022 polymorphisms based on publication year.

Polymorphism	Indexes	Allele	Homozygote	Heterozygous	Recessive	Dominant
PRM1 rs737008	R	0.152	0.639	0.573	0.572	0.066
	Adjusted R^2^	− 0.66	0.354	0.267	0.266	− 0.086
	*P*-value	0.620	**0.019**	**0.041**	**0.041**	0.831
PRM1 rs2301365	R	0.545	0.660	0.619	0.630	0.241
	Adjusted R^2^	0.209	0.365	0.306	0.322	− 0.060
	*P*-value	0.104	**0.038**	0.057	0.051	0.503
PRM2 rs1646022	R	0.225	0.698	0.267	0.358	0.534
	Adjusted R^2^	− 0.085	0.414	− 0.083	0.004	0.183
	*P*-value	0.561	**0.036**	0.522	0.344	0.139
PRM2 rs2070923	R	0.234	0.059	0.012	0.249	0.251
	Adjusted R^2^	− 0.103	− 0.163	− 0.166	− 0.094	− 0.093
	*P*-value	0.576	0.889	0.977	0.552	0.549

Allele: A vs. C, homozygous: AA vs. CC, heterozygous: AG vs. CC, recessive: AA + CA vs. CC, and dominant: AA vs. CA + CC, for PRM1 rs737008, PRM1 rs2301365, and PRM2 rs2070923 polymorphisms. Allele: C vs. G, homozygous: CC vs. GG, heterozygous: GC vs. GG, recessive: CC + GC vs. GG, and dominant: CC vs. GC + GG, for PRM2 rs1646022 polymorphism. Bold numbers indicate statistically significant differences.

### Sensitivity analysis

We excluded the studies with a deviation of HWE in the controls, including two studies^30,33^ for polymorphism of *PRM1* rs737008, six^25,29,30,36,38,39^ for *PRM2* rs1646022, and four^25,30,32,38^ for *PRM2* rs2070923. The results after excluding are presented in Table 6. Moreover, the sensitivity analysis based on “one study removed” and “cumulative analysis” on the previous analyses did not change the results and therefore confirmed the stability of the pooled data.

**Table 6:** Sensitivity analysis at the studies without deviation of HWE in the controls.

Polymorphism (number of studies)	Allele	Homozygote	Heterozygous	Recessive	Dominant
	OR (95% CI), *P*, I^2^, P_h_	OR (95% CI), *P*, I^2^, P_h_	OR (95% CI), *P*, I^2^, P_h_	OR (95% CI), *P*, I^2^, P_h_	OR (95% CI), *P*, I^2^, P_h_
PRM1 rs737008 (11)	0.96 (0.82, 1.14), 0.66, 51, 0.03	1.11 (0.86, 1.42), 0.42, 27, 0.19	0.95 (0.79, 1.14), 0.57, 43, 0.06	0.96 (0.81, 1.14), 0.65, 47, 0.04	1.07 (0.89, 1.27), 0.48, 16, 0.29
PRM2 rs1646022 (2)	1.20 (0.96, 1.48), 0.10, 0, 0.92	0.96 (0.59, 1.56), 0.87, 0, 0.67	1.05 (0.61, 1.80), 0.87, 67, 0.05	1.04 (0.63, 1.73), 0.88, 66, 0.05	0.98 (0.62, 1.56), 0.93, 0, 0.94
PRM2 rs2070923 (4)	0.94 (0.77, 1.14), 0.53, 12, 0.33	0.88 (0.58, 1.31), 0.52, 31, 0.22	0.80 (0.61, 1.06), 0.12, 11, 0.34	0.82 (0.63, 1.06), 0.12, 47, 0.13	0.97 (0.67, 1.41), 0.87, 0, 0.52

Allele: A vs. C, homozygous: AA vs. CC, heterozygous: AG vs. CC, recessive: AA + CA vs. CC, and dominant: AA vs. CA + CC, for PRM1 rs737008, and PRM2 rs2070923 polymorphisms. Allele: C vs. G, homozygous: CC vs. GG, heterozygous: GC vs. GG, recessive: CC + GC vs. GG, and dominant: CC vs. GC + GG, for PRM2 rs1646022 polymorphism.

### Publication bias

**Figure 6:**
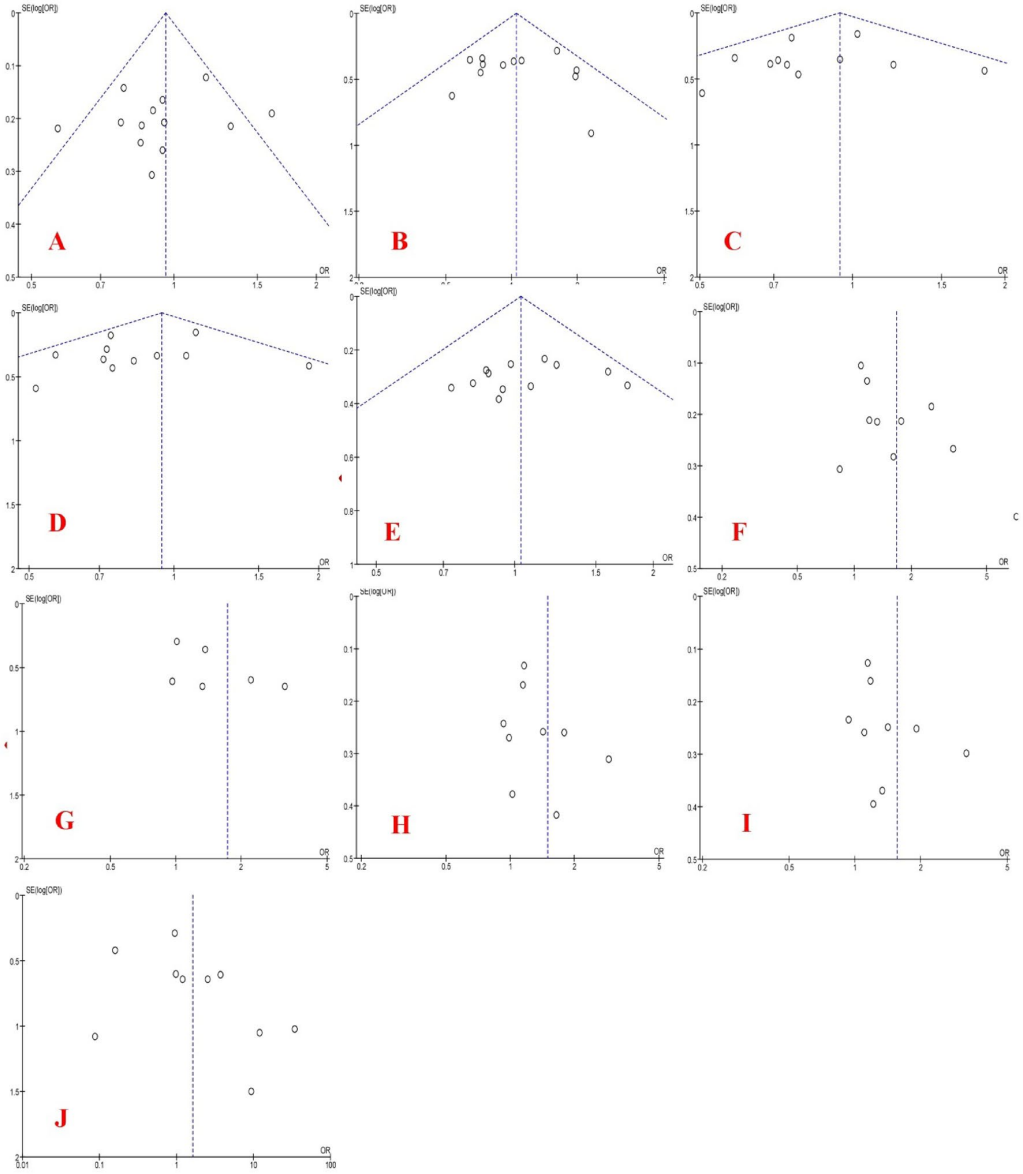
Funnel plots of PRM1 polymorphism based on five genetic models (allelic, homozygote, heterozygote, recessive, and dominant models, respectively): (**A**–**E**) for rs737008 and (**F**–**J**) for rs2301365.

**Figure 7:**
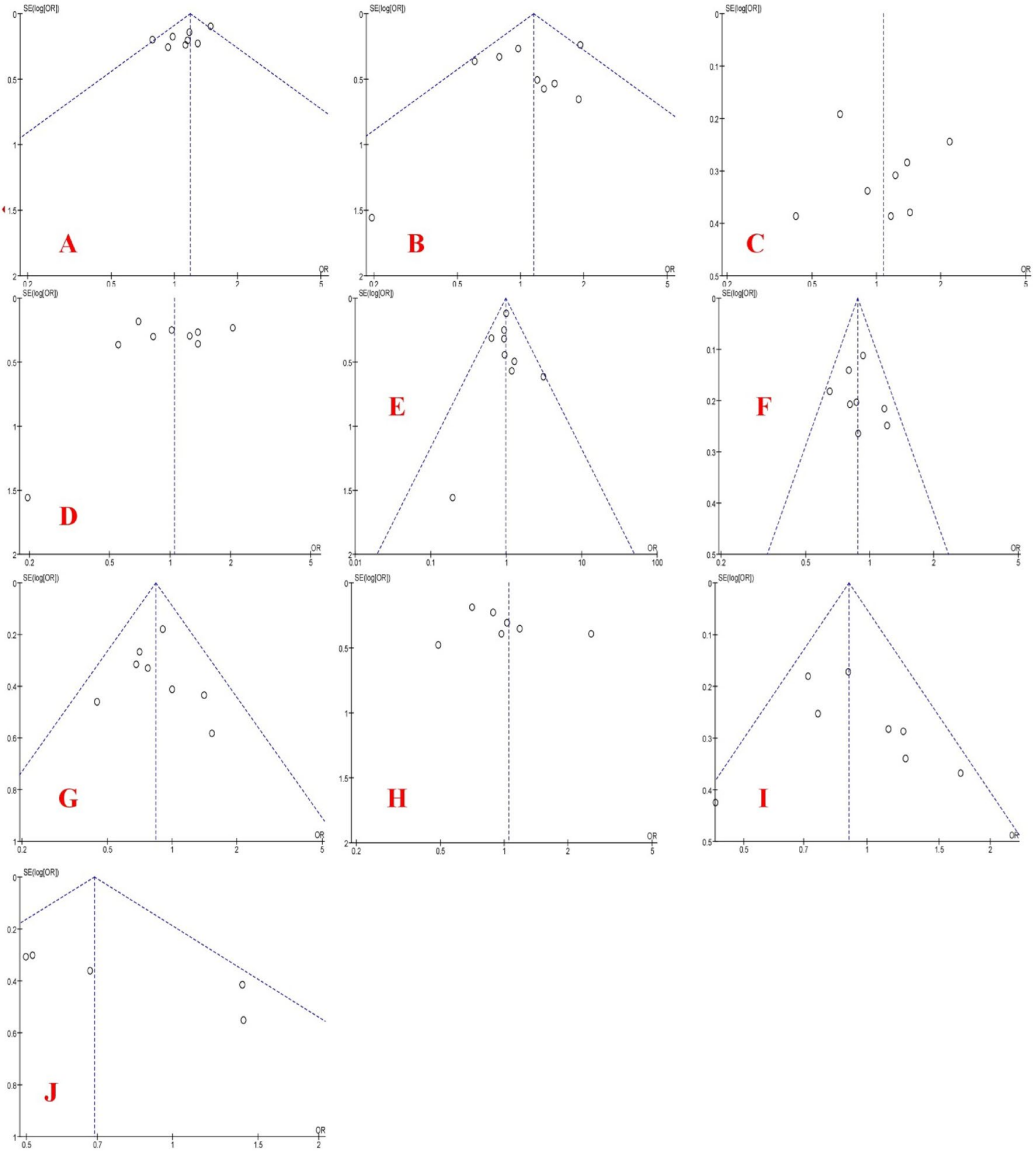
Funnel plots of PRM2 polymorphism based on five genetic models (allele, homozygote, heterozygote, recessive, and dominant models, respectively): (**A**–**E**) for rs1646022 and (**F**–**J**) for rs2070923.

The funnel plots of *PRM1* and *PRM2* polymorphisms based on five genetic models are shown in Figs. 6 and 7, respectively. As the results showed, Egger’s test revealed the publication bias for AA + CA vs. CC (P < 0.001) and AA vs. CA + CC models (*P* = 0.04) in *PRM1* rs737008 polymorphism and C vs. G model (*P* = 0.016) in *PRM2* rs1646022 polymorphism. In addition, Begg’s test revealed the publication bias for AA + CA vs. CC (*P* = 0.001) model in *PRM1* rs737008 polymorphism, CA vs. CC (*P* = 0.025) and AA + CA vs. CC models (*P* = 0.039) in *PRM1* rs2301365 polymorphism.

## Discussion

There is considerable empirical evidence to suggest that PRMs are necessary for male infertility and that PRM1 and PRM2 have a fundamental role in sperm chromatin density and spermatogenesis^40,41^ Any single nucleotide polymorphism in the coding and non-coding areas of *PRM1* and *PRM2* genes may cause significant abnormalities in their expression^9^. The changes in one set of genes and expression patterns impact the spermatogenesis process and its products, resulting in spermatogenesis dysfunction and leading to male infertility^42^. Nowadays, the findings on the association of *PRM* genes with male infertility are not fully convincing, and there are not sufficient studies on this topic^32^. A research confirmed that the expression of PRMs is uniquely related to the transcription/translation factors^43^. The present meta-analysis showed that *PRM1* rs737008 polymorphism was not associated with the risk of male infertility. *PRM1* rs2301365 and *PRM2* rs1646022 polymorphisms were associated with an elevated risk of male infertility and *PRM2* rs2070923 polymorphism had a protective role in infertile men. In addition, the subgroup analysis showed the effect of ethnicity, control source, and genotyping method on the association of PRM polymorphisms with the risk of male infertility. The results of meta-regression showed that publication year was a cofounding factor involved in the association between *PRM1* rs737008, *PRM1* rs2301365, and *PRM2* rs1646022 polymorphisms and susceptibility to male infertility. Although single nucleotide polymorphism of G197T that lead to arginine to serine conversion was required in highly protected clusters of arginine for normal DNA binding has been found in 10% of unrelated infertile cases whose sperms were phenotypically same as those from mice with PRMN deficiency^44^.

It has been shown that *PRM1* and *PRM2* variants are related to male infertility in both humans and animals^25,26^. In the animal model, reduction of PRM causes sperm morphology defects due to decreased motility and infertility as a result of haploid germ deficiency^45–47^. Using gene–gene interaction analysis, Jiang et al.^36^ examined twelve combined genotypes of *PRM* polymorphisms. Their results showed a significant association between the combined genotypes and male infertility. One study reported that sperm concentration, motility, and morphology significantly decreased in patients with an aberrant PRM ratio^48^. PRM protection is very important in mammals and minor alternations in the coding and non-coding regions of *PRM* genes may cause important abnormalities in the expression or maintenance of gene expression stability^9^. PRMs may act as a checkpoint for spermatogenesis, where abnormal PRM expression causes the induction of an apoptotic process that may explain the decrease in sperm production^12^. In addition, studies have shown that abnormal PRM expression is related to defective spermatogenesis^12^. There is some evidence that *PRM* mutations or polymorphisms may induce alternations at the protein level and their composition in sperm chromatin, resulting in sperm deficiency^46,47^. Semen quality decreases with age and characteristic molecular changes occur during aging (increased damage of sperm DNA, sperm infection changes, and plasma miRNA profile changes). In addition, the logistic regression models have illustrated an association between age and semen parameters^49^.

As the present meta-analysis demonstrated, ethnicity, control source, and genotyping method of *PRM* polymorphisms are important and may contribute to the difference in susceptibility to male infertility. A meta-analysis^17^ reported an association between *PRM1* rs2301365 polymorphism and the risk of male infertility in the Caucasians, not in the Asians. As in our meta-analysis, there was an elevated risk of male infertility for *PRM1* rs2301365 polymorphism only in Caucasians and for *PRM2* rs1646022 polymorphism only in Asians. In addition, there was significantly a decreased risk of *PRM1* rs737008 in population-based controls, elevated risk of *PRM1* rs2301365 and *PRM2* rs1646022 in hospital-based controls. Also, with regards to method, an elevated risk of *PRM1* rs2301365 and a decreased risk of *PRM2* rs2070923 in PCR-based method and an elevated risk of *PRM2* rs1646022 in Mass ARRAY method. It is noteworthy that the expression of genes, environmental factors, and spermatogenesis disorder can play an important role in male sterility^9^. Another possible reason for these inconsistent findings can be a particular selection of the clinical subtypes of male infertility and *PRM1* and *PRM2* variations in different populations examined^9^. Therefore, existence of heterogeneity among studies may be due to the differences genotyping method, clinical subtypes of male infertility, ethnicity, publication year, control source, and even number of recruited patients^38^.

This meta-analysis had two significant limitations. First, the clinical data such as age, abstinence time, serum hormone index, and semen quality and parameters were not analyzed due to lack of information. Second, the meta-analysis did not evaluate the gene–gene and gene-environment interactions due to lack of information in the published studies.

## Conclusions

The present meta-analysis evaluated four *PRM* polymorphisms (*PRM1* rs737008, *PRM1* rs2301365, *PRM2* rs1646022, and *PRM2* rs2070923). The results showed *PRM1* rs2301365 and *PRM2* rs1646022 polymorphisms were associated with an elevated risk of male infertility and *PRM2* rs2070923 polymorphism had a protective role in infertile men. In addition, ethnicity, control source, and genotyping method impacted the *PRM* polymorphisms and susceptibility to male infertility. Based on the results, the future studies need to evaluate these polymorphisms in a large number of participants in various areas, with an emphasis on environmental factors, interactions, age, method, and selection of controls (deviation of HWE and source).
